# Supplementary material for: Impacts of structural properties of myosin II filaments on force generation
Source: eLife. 2025 Aug 13;14:RP105236. doi: 10.7554/eLife.105236 (PMC12349899; doi:10.7554/eLife.105236)
Supplement: Supplementary file 1. [file elife-105236-supp1.docx]

**Supplementary File 1 List of parameters used in the model.**

| **Symbol** | **Definition** | **Value** |
| --- | --- | --- |
| *r*_0,A_ | Equilibrium length of the actin segment | 1.4×10^-7^ [m] |
| *r*_c,A_ | Diameter of the actin segment | 7.0×10^-9^ [m] |
| *θ*_0,A_ | Equilibrium angle of F-actin | 0 [rad] |
| *κ*_s,A_ | Extensional stiffness of F-actin | 1.69×10^-2^ [N/m] |
| *κ*_b,A_ | Bending stiffness of F-actin | 2.64×10^-19^ [N∙m] |
| *r*_0,ACP_ | Equilibrium length of the ACP segment | 2.35×10^-8^ [m] |
| *r*_c,ACP_ | Diameter of the ACP segment | 1.0×10^-8^ [m] |
| *θ*_0,ACP_ | Equilibrium angle of ACP | 0 [rad] |
| *κ*_s,ACP_ | Extensional stiffness of the ACP | 2.0×10^-3^ [N/m] |
| *κ*_b,ACP_ | Bending stiffness of ACP | 1.04×10^-19^ [N∙m] |
| *r*_0,M1_ | Equilibrium length of the motor backbone segment | 42-138 [nm] |
| *r*_c,M_ | Diameter of the motor backbone segment | 1.0×10^-8^ [N/m] |
| *θ*_0,M_ | Equilibrium angle of the motor backbone | 0 [rad] |
| *κ*_s,M1_ | Extensional stiffness of the motor backbone | 1.69×10^-2^ [N/m] |
| *κ*_b,M_ | Bending stiffness of the motor backbone | 5.07×10^-18^ [N∙m] |
| *r*_0,M2_ | Equilibrium length 1 of the motor arm | 1.35×10^-8^ [m] |
| *r*_0,M3_ | Equilibrium length 2 of the motor arm | 0 [m] |
| *κ*_s,M2_ | Extensional stiffness 1 of a motor arm | 1.0×10^-3^ [N/m] |
| *κ*_s,M3_ | Extensional stiffness 2 of a motor arm | 1.0×10^-3^ [N/m] |
| *k*_n,A_ | Nucleation rate constant in network simulations | 1.0×10^-6^ [μM^-1^s^-1^] |
| *k*_p,A_ | Polymerization rate constant in network simulations | 6.0×10^4^ [μM^-1^s^-1^] |
| *N*_h_ | Number of myosin heads represented by a motor arm | 8 |
| *N*_a_ | Number of arms in a single motor | 4-48 |
| *κ*_r,A_ | Strength of the repulsive force | 1.69×10^-3^ [N/m] |
| *N*_M_ | Number of motors in the system | 1-2,000 |
| Δt | Time step | 1.15×10^-5^ [s] |
| *μ* | Viscosity of medium | 8.6×10^-1^ [kg/m∙s] |
| *k*_B_*T* | Thermal energy | 4.142×10^-21^ [J] |
