# Supplementary material for: Impacts of structural properties of myosin II filaments on force generation
Source: eLife. 2025 Aug 13;14:RP105236. doi: 10.7554/eLife.105236 (PMC12349899; doi:10.7554/eLife.105236)
Supplement: Supplementary file 2. — Note that we used slightly different values for F0, d, and km from those in the literature. [file elife-105236-supp2.docx]

**Supplementary File 2 List of parameter values used for adopting the “parallel cluster model”.** Note that we used slightly different values for *F*_0_, *d*, and *k*_m_ from those in the literature.

| **Symbol** | **Definition** | **Value** |
| --- | --- | --- |
| *k*_01_ | A rate from unbound to weakly bound state | 40 [s^-1^] |
| *k*_10_ | A rate from weakly bound to unbound state | 2 [s^-1^] |
| *k*_12_ | A rate from weakly bound to post-power-stroke state | 1,000 [s^-1^] |
| *k*_21_ | A rate from post-power-stroke to weakly bound state | 1,000 [s^-1^] |
| *k*_20_ | A rate from post-power-stroke to unbound state | 20 [s^-1^] |
| *F*_0_ | Force dependence | 5.04×10^-12^ [N] |
| *E*_pp_ | Free energy bias toward the post-power-stroke state | -60×10^-21^ [J] |
| *E*_ext_ | External energy contribution | 0 [J] |
| *d* | Step size | 7×10^-9^ [m] |
| *k*_m_ | Spring constant of the neck linkers | 1×10^-3^ [N/m] |
